# Distinct microbial communities across a climatically versatile summit in the Lesotho highlands

**DOI:** 10.1002/ece3.9891

**Published:** 2023-03-15

**Authors:** Jasmin Patel, Stefan Grab, Pieter De Maayer

**Affiliations:** ^1^ School of Molecular and Cell Biology, Faculty of Science University of the Witwatersrand Johannesburg South Africa; ^2^ School of Geography, Archaeology and Environmental Studies, Faculty of Science University of the Witwatersrand Johannesburg South Africa

**Keywords:** community structure, Lesotho highlands, microbial diversity, microclimatic factors

## Abstract

Most studies investigating the effects of climatological factors on microbial community composition and diversity focus on comparisons of geographically distinct environments (e.g., cold vs hot deserts) or across various temporal scales. Mountain regions provide unique environments to explore relationships between various environmental factors and soil microorganisms given their range of microclimatic conditions and vegetation types. This study investigated micro‐topographically (i.e., north‐/south‐facing slope aspects and flat plateau between them) controlled microbial diversity and community structures across a Lesotho mountain summit. Amplicon sequence analysis revealed that the north‐ and south‐facing slopes were dominated by more Proteobacteria and Bacteroidetes, while the plateau was dominated by more Acidobacteria. Fungi from the phylum Chytridiomycota more strongly dominated the plateau and the north‐facing slope than the south‐facing slope. Slope aspect, through its direct influence on air and soil micro‐climatology and plant diversity, significantly affects bacterial and fungal community structures at this location. These results provide original insight into soil microbial diversity in the Lesotho highlands and offer an opportunity to project the likely response of soil microorganisms to future climate warming in highly variable mountain environments such as the Lesotho highlands.

## Graphical abstract

A peak in the Lesotho highlands sampled in this study showed extensive differences in temperature and relative humidity over a very short spatial scale. Here, by means of amplicon sequence analysis, we show that these factors significantly impact soil microbial ecology. This can serve as a primer to study climate change across short spatial scales.

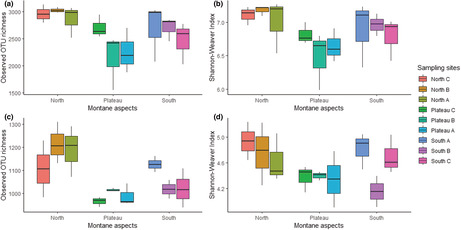

## 1 INTRODUCTION

Approximately 24% of the Earth's land mass is covered by mountains, which comprise of diverse ecosystems owing to a range of climatic types including tropical, temperate, and cryogenic (García‐Llamas et al., 2019; United Nations, 1992). Mountains also provide humanity with essential resources and services, such as water, minerals, energy, carbon sequestering, agricultural resources, recreation, and tourism (García‐Llamas et al., 2019; Lu, 2021; Martín‐López et al., 2019; United Nations, 1992). In addition to climate warming, mountain environments are characterized by strong topographical diversity through elevation, slope gradients, and slope aspect (directional orientation of slopes) (Singh, 2018). The complex combination of such topographic factors drives climatic and environmental variability in such settings (Donhauser & Frey, 2018). Slope aspect influences solar radiation intensity and duration (Måren et al., 2015), creating spatial variations in air and soil temperatures, soil moisture regimes (Jakšić et al., 2021; Yang et al., 2020), soil properties (organic matter content, nutrient availability, and soil depth and texture) (Yang et al., 2020), and vegetation growth and distribution (Badano et al., 2005; Gong et al., 2008).

In the Southern Hemisphere (SH), north‐facing slopes are oriented more directly toward the sun and receive greater intensity and duration of solar radiation than do opposing south‐facing slopes (Codrington, 2005; Gilliam et al., 2014). High elevation north‐facing slopes in the SH thus experience warmer climatic conditions with less shade and faster snow melt than south‐facing slopes, especially during winter months (Gilliam et al., 2014; Jakšić et al., 2021). Consequently, soils retain less moisture, and vegetation is generally more tolerant to warmer and drier conditions on north‐facing slopes than on opposing south‐facing slopes (Måren et al., 2015; Paudel & Vetaas, 2014). In contrast, south‐facing slopes retain moisture for longer and are cooler, supporting more shade and moisture‐tolerant vegetation (Erdős et al., 2012; Pandita et al., 2019). These variations in species composition and richness of vegetation across mountain slope aspects also affect the quality and quantity of litter and organic matter available within the soil (Jakšić et al., 2021; Xue et al., 2018). Warmer north‐facing slopes have less soil organic matter due to greater mineralization rates and sparse vegetation patterns, compared to the cooler south‐facing slopes (Jakšić et al., 2021; Mohammad, 2008). This ultimately shapes and determines the number and type of soil microorganisms that inhabit mountain soils, causing either an increase or decrease in decomposition rates of organic matter and enzyme activity across a particular slope (Xue et al., 2018), underpinning Baas Bekings’ statement “everything is everywhere, but, the environment selects” with regard to microbial diversity and biogeography (Baas‐Becking, 1934).

Given that soil microorganisms are primarily responsible for processes such as nutrient cycling, decomposition of organic matter, and the formation of symbiotic relationships with higher organisms, these microorganisms are essential for ecosystem functioning and structure (Gilliam et al., 2014; Ma et al., 2004). While several studies determining the impact of mountain slope aspect on living organisms have focused on vegetation patterns (e.g., (Måren et al., 2015; Singh, 2018; Yang et al., 2020)), studies on the impact of (micro‐) topography on soil microorganisms have only recently been forthcoming (Singh, 2018; Zeng et al., 2014). Researchers in several mountain ranges across China, Israel, New Zealand, and Europe have indicated that slope aspect plays a significant role in altering bacterial and fungal community structures (Adamczyk et al., 2019; Chu et al., 2016; Liu et al., 2017; Moroenyane et al., 2020; Wu et al., 2017; Xue et al., 2018). Some studies have shown bacterial community composition to be greater across warmer slopes, while fungal community composition is greater on cooler slopes (Chu et al., 2016; Wu et al., 2017). In contrast, other researchers have found a greater bacterial community composition across cooler slopes and greater fungal community composition across warmer slopes (Liu et al., 2017; Moroenyane et al., 2020). Despite such contradictory findings, differences noted in community composition have been linked to differences in vegetation patterns, soil properties and conditions, and environmental factors caused by slope aspect, all of which play a crucial role in shaping soil microbiomes (Chu et al., 2016; Liu et al., 2017; Moroenyane et al., 2020; Wu et al., 2017).

Mountain environments and their inhabited species are severely impacted by climate warming, and thus, it is the cause for concern (Sauer et al., 2011). Climate warming in these environments has already caused mean annual temperatures to increase 1.24 times faster in regions >500 meters above sea level (m.a.s.l.) compared to lower lying regions (i.e., <500 m.a.s.l.) over the period 1961–2010 (Wang et al., 2020). Soil microorganisms on mountains react to such climate warming through various responses, including changes in phenology, physiology, and community structure and distribution across habitats (Di Nuzzo et al., 2021; Sauer et al., 2011). Increasing temperatures also lead to an upward‐elevational shift in suitable habitats, thereby causing compositional changes in species of soil microorganisms across mountains (Elsen & Tingley, 2015). However, due to topographic and land cover constraints toward mountain summits, the risk of extinction of soil microorganism species increases as suitable habitat availability decreases and competition for resources increases (Di Nuzzo et al., 2021).

**FIGURE 1:**
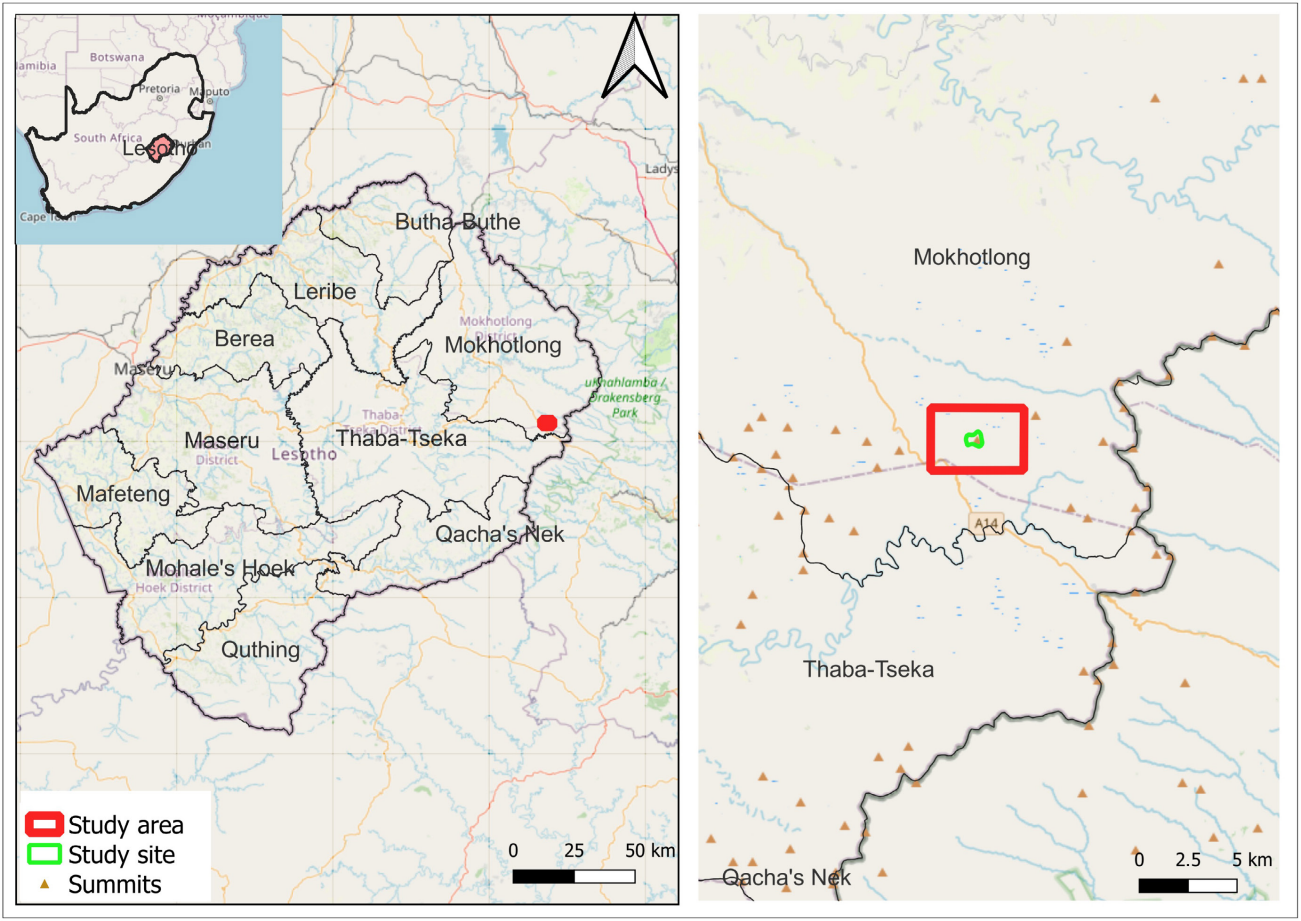
Geographical location of the study site in Lesotho (map adapted from QGIS, n.d.).

To date, little is known about the influence of topography and associated microclimates on soil microbial diversity in southern Africa's highest mountain region, the Drakensberg (Figure 1). To this end, we explore bacterial and fungal diversity across a high (~3350 m.a.s.l.) mountain peak located near Kotisephola Pass in the Drakensberg, Lesotho, with the specific aim to establish any possible association between slope aspect‐controlled soil temperatures/humidity and the overall microbiome. We aimed to test the following hypothesis: due to variations in vegetation zonation and microclimates associated with topographic location (i.e., slope aspect), bacterial and fungal community structures will vary across small spatial scales over mountain summits.

## 2 EXPERIMENTAL PROCEDURES

### 2.1 Study area

The mountainous kingdom of Lesotho is the highest region in southern Africa, located between the latitudes 28°30′ to 30°40′ S and the longitudes 27°00′ to 29°30′ E (Grab et al., 2017). Lesotho's total land area of 30,355 km^2^ comprises mostly high mountains and deep valleys, with all its land located over 1400 m.a.s.l. (Roberts et al., 2013). The climate varies from semi‐arid to sub‐humid, with generally mild‐cool and wet summers, and cold and dry winters (Grab & Nash, 2010). Frost and several light‐to‐moderate snowfalls occur during the cooler seasons from May to September (Grab et al., 2017; Sene et al., 1998).

**FIGURE 2:**
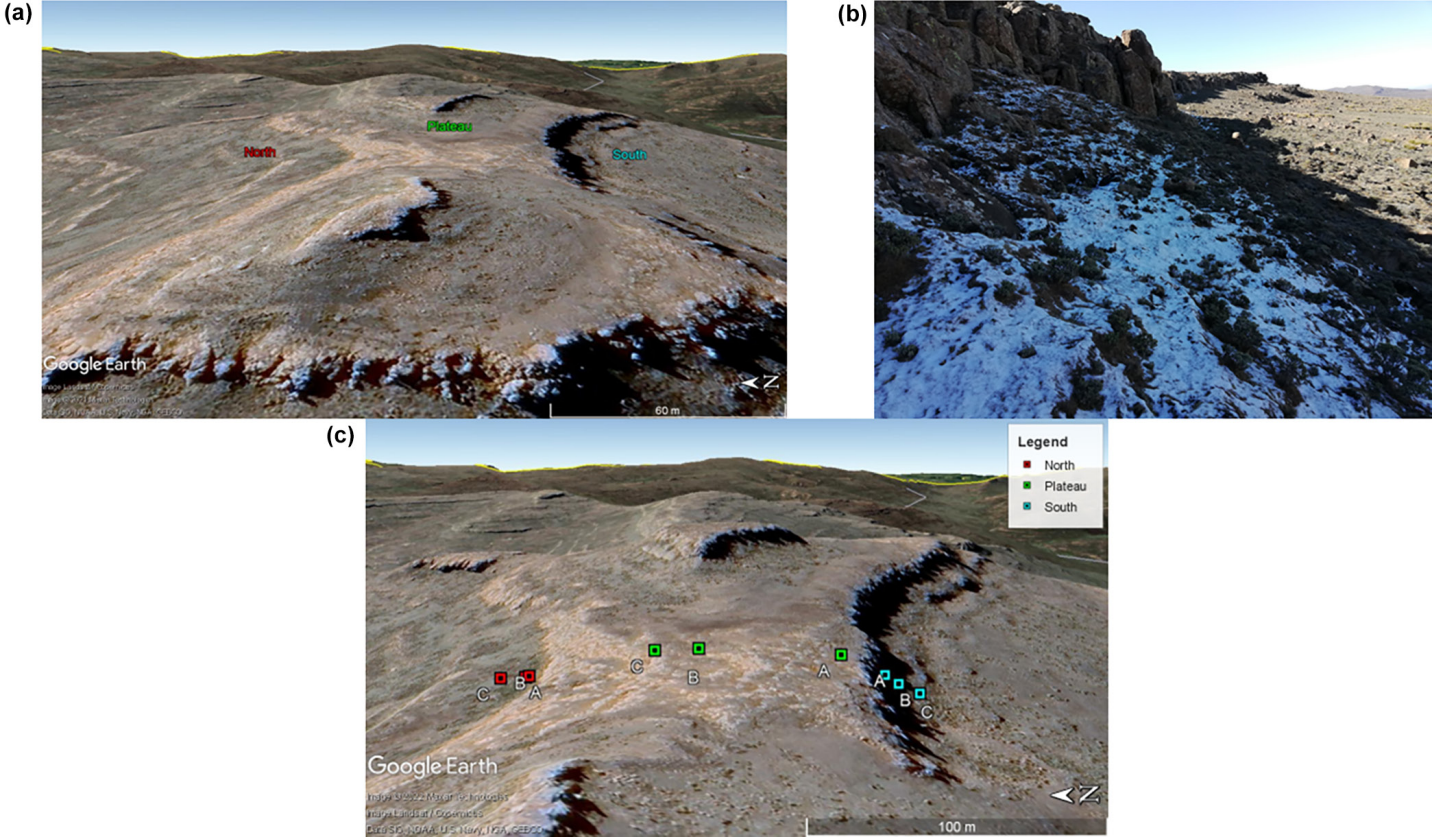
Photographic and geographic layout of the study site in Lesotho. (a) Three sampling locations across the summit. (b) Layout of the rock scarp across the south‐facing slope. (c) Distribution of the sampling sites across the plateau and the north‐ and south‐facing slopes. The image also provides a representation of rock‐scarp morphology along the north‐ and south‐facing slopes (map images adapted from Google Earth, n.d.).

Our study was conducted along a slope transect, which included north‐facing (N), summit plateau (P), and south‐facing (S) slope positions (Figure 2a). The chosen summit is located near Kotisephola Pass, inland of the infamous Sani Pass which marks a border between South Africa and Lesotho (Figure 1). The region falls between the longitudinal coordinates 29°30′46 to 29°30′52 south and the latitude 29°13′09 east. Sampling was undertaken over an elevational range of 3330 to 3350 m.a.s.l. Three sampling sites (A, B, and C) were selected along P, N, and S, respectively (Figure 2c), providing nine distinct sampling points (Table 1). The three sampling sites (A, B, and C) along the P, N, and S, were, respectively, selected based on variations in topographic position (i.e., slope aspect) and associated differences in vegetation characteristics and microclimatic conditions. The underlying basaltic lithology is uniform across the slopes.

**TABLE 1:** Overview of the sampling sites and their location on the selected summit.

Primary site	Secondary site	Site location	Replicates	Elevation (m.a.s.l.)	Latitude and longitude
South	South A	Top of the slope	SA1	3338	29°30′51.9″ S 29°13′09.3″ E
			SA3	3336	29°30′52.1″ S 29°13′09.2″ E
			SA4	3336	29°30′52.0″ S 29°13′09.4″ E
	South B	Middle of the slope	SB1	3332	29°30′52.2″ S 29°13′09.3″ E
			SB2	3335	29°30′52.1″ S 29°13′09.5″ E
			SB4	3334	29°30′52.3″ S 29°13′09.3″ E
	South C	Bottom of the slope	SC2	3334	29°30′52.6″ S 29°13′09.3″ E
			SC3	3330	29°30′52.7″ S 29°13′09.0″ E
			SC4	3329	29°30′52.7″ S 29°13′09.1″ E
Plateau	Plateau A	Southern end of summit	PA1	3348	29°30′51.4″ S 29°13′09.1″ E
			PA3	3348	29°30′51.2″ S 29°13′09.2″ E
			PA4	3347	29°30′51.3″ S 29°13′09.4″ E
	Plateau B	Midway of plateau	PB1	3349	29°30′49.5″ S 29°13′09.2″ E
			PB2	3349	29°30′49.4″ S 29°13′09.3″ E
			PB3	3349	29°30′49.6″ S 29°13′09.4″ E
	Plateau C	Northern end of summit	PC1	3349	29°30′48.6″ S 29°13′09.4″ E
			PC3	3348	29°30′48.4″ S 29°13′09.5″ E
			PC4	3348	29°30′48.4″ S 29°13′09.4″ E
North	North A	Top of the slope	NA1	3335	29°30′47.5″ S 29°13′09.2″ E
			NA2	3338	29°30′47.5″ S 29°13′09.4″ E
			NA4	3335	29°30′47.3″ S 29°13′09.2″ E
	North B	Middle of the slope	NB1	3335	29°30′47.1″ S 29°13′09.3″ E
			NB2	3335	29°30′47.1″ S 29°13′09.3″ E
			NB3	3335	29°30′47.2″ S 29°13′09.3″ E
	North C	Bottom of the slope	NC2	3333	29°30′46.7″ S 29°13′09.6″ E
			NC3	3333	29°30′46.6″ S 29°13′09.6″ E
			NC4	3332	29°30′46.6″ S 29°13′09.5″ E

### 2.2 Soil sample collection

The three primary sampling areas along the north‐facing slope, plateau, and south‐facing slope were distinct in terms of vegetation growth and patterns, microclimatic conditions, slope topography (rock scarp), and slope aspect. The south‐facing slope has a relatively high (~5–6 m) and near vertical (80–90°) rock scarp, which results in long‐lasting shade on slopes immediately below the scarp, and consequently permits snow accumulation through a snow‐fencing effect and also preservation through protection from direct insolation. Snow patches may last from late May to early October (Figure 2b), but is variable between years. Vegetation closest to the rock scarp (SA ~5.50 m distance) includes low *Helichrysum* shrubs, which then gradually incorporate a higher abundance of tall *Merxmuellera drakensbergensis* tussock grass with increasing distance downslope from the rock scarp (SB ~13.20 m and SC ~26.80 m distance). In contrast, the north‐facing rock scarp is more shallow in gradient (50–60°) and does not provide for sharing on the slope below. Here, snow longevity is limited to 2 or 3 days post snowfalls.

The plateau is exposed to enhanced levels of solar radiation with no rock scarp present to provide shade. The plateau was sampled 19.82 m (PA) away from the southern end of the summit edge (SA) and 52.50 m (PC) distance from the northern end of the summit edge (NA) (Figure 2c). PA was located 57.50 m distance from PB, and PC was located 17.98 m distance from PB. Regolith cover was deepest (>0.5 m) on the south‐facing slope, of intermediate depth (0.1–0.5 m) on the north‐facing slope, and generally absent to thin (<0.1 m) across the plateau.

These topographic factors consequently yield strongly contrasting abiotic dynamics on soil‐covered slopes across our transect. Distance of sampling locations from the north‐facing slope rock scarp mirrored that of the south‐facing slope, measuring 5.50 m (NA), 13.20 m (NB), and 26.80 m (NC) downslope from the rock scarp (Figure 2c). Vegetation patterns also varied down the north‐facing slope. Our sampling/monitoring site closest to the rock scarp (NA) consisted mainly of tall tussock grass, which then gradually incorporated a higher abundance of low shrubs at sites further away from the rock scarp (NB and NC).

The presence of fecal matter from livestock grazing was also visible across the plateau and the north‐ and south‐facing slopes. Fecal matter was at a greater visible abundance across the north‐facing slope along the top (NA), middle (NB), and lower sites (NC), while across the south‐facing slope a greater abundance was noted toward the middle (SB) and lower sites (SC). The plateau had considerably lower visible scattering of fecal matter across all three sites.

Soil samples were collected in replicates of four from a 4 m × 4 m plot at each of the nine locations. Plant material, debris, stones, and pebbles were scraped from the sampling site, and soil samples were collected from a depth of ~5 cm below the surface. Soil samples were transported back to the laboratory and were stored at 4°C until processed.

### 2.3 Soil climate data collection

Hydrochron iButtons (DS1923 Maxim, San Jose, U.S.) were installed in the center of each of the 4 m × 4 m plots across the nine sampling locations during initial soil collection in May 2019. The iButtons were glued onto a wooden peg to prevent movement in the soil and set at a depth of 5 cm below the surface. The iButtons measured mean, maximum, and minimum soil temperature and relative humidity (RH) at 2‐h intervals from 01:59 on 15 May, 2019, to 15:59 on 19 April, 2020. Data from the iButtons were retrieved using ColdChain ThermoDynamics v 4.9 (Fairbridge Technologies, Sandton, SA) and subsequently analyzed for comparison with soil microbiome data.

### 2.4 DNA extraction, PCR amplification, and high‐throughput sequencing

Total soil DNA was extracted from ~0.4 g soil aliquots using the MoBio Powersoil DNA isolation kit (Qiagen), as per the manufacturer's instructions. DNA was quantified using the Nanodrop (Thermo Fisher Scientific) and was stored at −4°C until further use.

From the four replicates of soil samples collected at each of the nine sampling locations, a total of three random sample replicates per sampling location were selected to achieve a final batch of 27 samples (Table 1). These samples were then sent to Molecular Research LP (www.mrdnalab.com) for amplicon sequencing. Partial bacterial 16 S rRNA amplicons were generated using the 27F (5′‐AGRGTTTGATCMTGGCTCAG‐3′) and 519R (5′‐GTNTTACNGCGGCKGCTG‐3′) primers, which target the hypervariable regions V1–V4 and partial fungal ITS2 amplicons were generated using the ITS1F (5′‐CTTGGTCATTTAGAGGAAGTAA‐3′) and ITS4 (5′‐TCCTCCGCTTATTGATATGC‐3′) primers which target the ITS2 hypervariable region (Valverde et al., 2016). PCR amplification was performed using the HotStarTaq Plus Master Mix Kit (Qiagen) as per the manufacturer's instructions. Amplicon products were pooled, based on their molecular weight and DNA concentration, and thereafter purified using Ampure XP beads. Following library preparation with the pooled and purified PCR products with the Illumina DNA Prep Kit (Illumina), amplicon sequencing was performed using the Illumina MiSeq platform with the paired‐end read approach (2 × 150 bp).

### 2.5 Sequence processing

The raw sequence data were processed using the MR DNA analysis pipeline (www.mrdnalab.com). Initial processing of the reads included joining of paired‐end reads, removal of barcodes, removal of reads less than 150 base pairs in length, quality filtering of trimmed sequences, and denoising of sequences in which sequence errors were rectified. Subsequently, chimeric sequences were removed using UCHIME (Edgar et al., 2011). The trimmed and filtered reads were clustered according to their Operational Taxonomic Units (OTUs) on the basis of 97% sequence identity using USEARCH (Edgar, 2010). The OTUs were taxonomically classified using BLASTn against a curated database which was derived from the Ribosomal Database Project (www.rdp.cme.msu.edu) and NCBI (www.ncbi.nlm.gov).

### 2.6 Statistical analysis

The processed sequences were analyzed using R v 3.6.1 (R Foundation for Statistical Computing; http://www.R‐project.org) and RStudio (RStudio Team, 2021). First, non‐bacterial and non‐fungal OTUs were removed from the dataset after which singleton sequences were removed, and the datasets were rarefied to standardize sample sizes (Valverde et al., 2016). Prior to rarefaction, two fungal sample replicates, SB2 (7599 sequences) and NC3 (19,403 sequences), were excluded from the analysis due to low numbers of sequences before singleton removal. The bacterial 16S rRNA reads were rarefied to 22,818 reads, whereas fungal ITS sequences were rarefied to 45,378 reads, representing the minimum number of sequences remaining in a single sample after the removal of low abundance counts.

Bacterial and fungal α‐diversity was estimated using observed OTU richness and the Shannon‐Weaver diversity index. Data distribution of α‐diversity indices was determined using the Shapiro–Wilk test for normality. The relationship between these α‐diversity indices and the geographical factor of slope aspect (categorical data) was then tested using the Kruskal–Wallis test. The Kruskal–Wallis test which is the non‐parametric equivalent test of analysis of variance (ANOVA) and Wilcoxon rank‐sum test with *p*‐values adjusted using the false discovery rate (fdr) correction method were performed for bacteria (non‐normally distributed) and ANOVA and Tukey's Honest Significant Difference test (Tukey's HSD) for fungi (normally distributed). The relationship between the α‐diversity indices and abiotic (soil temperature and RH) factors (continuous data) was tested using a general linear model following Quasipoisson distribution and the Gaussian distribution for bacteria and fungi, respectively, using the function *glm* from STAT (R Core Team, 2021). The relationship of interactions between the abiotic (soil temperatures and RH) and geographical factor (slope aspect) on bacterial and fungal α‐diversity indices was also determined using general linear mixed models by applying the function *glm*.

Bacterial and fungal β‐diversity was analyzed using Bray–Curtis dissimilarity based on the relative abundance of OTUs. The impact of abiotic and geographical factors on community structures was determined using permutational ANOVA (PERMANOVA) with 1000 permutations. Pairwise β‐diversity indices were calculated using the *vegdist* function and then the *adonis* function from vegan (Oksanen et al., 2020). Similarities between community structures were further visualized using non‐metric multidimensional scaling (nMDS) with Bray–Curtis distance. The dispersion of the groups was further determined using the *betadisp* function.

Differential abundance testing was conducted using the *DESeq* function from DESeq2 (Love et al., 2014). The targets used included the plateau and the south‐facing slope, while the north‐ and south‐facing slopes were set as the base levels for comparisons, respectively.

## 3 RESULTS

### 3.1 Soil microclimatic conditions

Temperature data across the slopes indicate that the south‐facing slope was significantly colder than the north‐facing slope and the plateau, both annually and seasonally (Table A1). Over the entire recording period, mean soil temperatures (5 cm depth) measured as follows: south‐facing slope = 5.92°C, plateau = 8.34°C, and north‐facing slope = 9.54°C. Temperatures contrasted most strongly during the winter months (June–August 2019) when the mean soil temperatures were as follows: south‐facing slope = −1.84°C, plateau = 3.58°C, and north‐facing slope = 5.38°C. Sampling sites on the north‐ and south‐facing slopes closest to the rock scarp (NA and SA) experienced the most extreme soil temperatures, with a maximum of 39.55°C on the north‐facing slope (maximum soil temperature for SA = 26.13°C) and a minimum of −10.06°C on the south‐facing slope (lowest soil temperature for NA = −6.55°C). In addition, the north‐facing slope experienced a large temperature range (46.10°C) between 21 July and 27 August 2019, while it only ranged by 12.09°C on the south‐facing slope over this period. Furthermore, the largest difference in temperature of 29.06°C across two different slopes at the same time (08:00 on 13 September 2019) was noted between the top of the north‐facing slope (NA) and the bottom of the south‐facing slope (SC). Such large variability in temperatures (>20°C) at a given point in time was most prominent between the top of the north‐facing slope (NA) and all three sampling locations across the south‐facing slope (SA, SB, and SC) over several days between May and October 2019.

Analysis of RH for soils across the slopes indicated that the south‐facing slope had a significantly higher mean RH (99.74%) than the north‐facing slope (88.69%) and plateau (85.07%), annually and seasonally (Table A1). Maximum RH reached 99.74% on the south‐facing slope, 88.69% on the north‐facing slope, and 111.85% on the plateau during mid‐autumn (April 2020). Notably, water ponding on the surface and super‐saturation of soils is not uncommon on such plateau surfaces given the shallow regolith cover (per observation by S. Grab). Exceptionally high RH values were also recorded on the north‐ and south‐facing slopes (111.07% and 110.61%, respectively) during late summer (February) and early autumn (April) of 2020. Such abnormally high RH readings are associated with the response of iButtons to stressors as iButtons function normally at a range of 50–100%. Stressors include the formation and deposition of water films over the device during periods of supersaturation through occurrences of heavy rain and snowfall.

### 3.2 Taxonomic classification of bacterial and fungal communities

**FIGURE A1:**
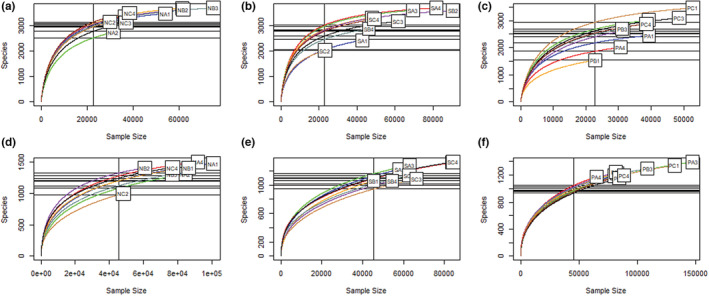
Bacterial and fungal rarefaction curves. Rarefaction curves represent the rarefied samples of bacterial OTUs to 22,818 and fungal OTUs to 45,378 on the north‐facing slope (a, d), on the plateau (c, f), and on the south‐facing slope (b, e), respectively.

The amplicon datasets derived from soil samples were first rarefied to a total of 22,818 and 45,378 reads for bacteria and fungi, respectively. These reflected the lowest counts present in sample PB1 (center of plateau) for bacteria and SB1 (middle of south‐facing slope) for fungi, respectively. Rarefaction curves generated for each of the samples for both the bacterial and fungal counterparts gradually reached a leveled state, which indicates that the process used to standardize sample sizes preserved the integrity of the samples (Figure A1). This represents the structure of all fungal and bacterial communities found in soils across the three slopes.

Following quality filtering, singleton removal, and sample rarefaction, a total of 1,750,536 sequences were retained for the 27 samples. The number of sequences obtained for the bacterial 16 S rRNA gene (616,086 sequences) was lower than that obtained for the fungal ITS assay (1,134,450 sequences). Bacterial sequences were classified into 4443 distinct OTUs, while fungal sequences were classified into 1961 distinct OTUs, respectively.

The bacterial communities across all sample sites were classified into 22 phyla, 52 classes, and 339 genera. Bacterial phyla were predominated by Proteobacteria (28%; mostly Alphaproteobacteria – 10% of total, Betaproteobacteria – 8%, and Deltaproteobacteria – 8%), Acidobacteria (21%, mainly from the class Acidobacteria – 19% of total), Verrucomicrobia (15%), Actinobacteria (11%), and Bacteroidetes (8%). The most abundant bacterial genera across the three slope aspects included *Acidobacterium* (17%), *Prosthecobacter* (8%), *Solirubrobacter* (4%), *Sphingobacterium* (3%), and *Conexibacter* (3%).

**FIGURE 3:**
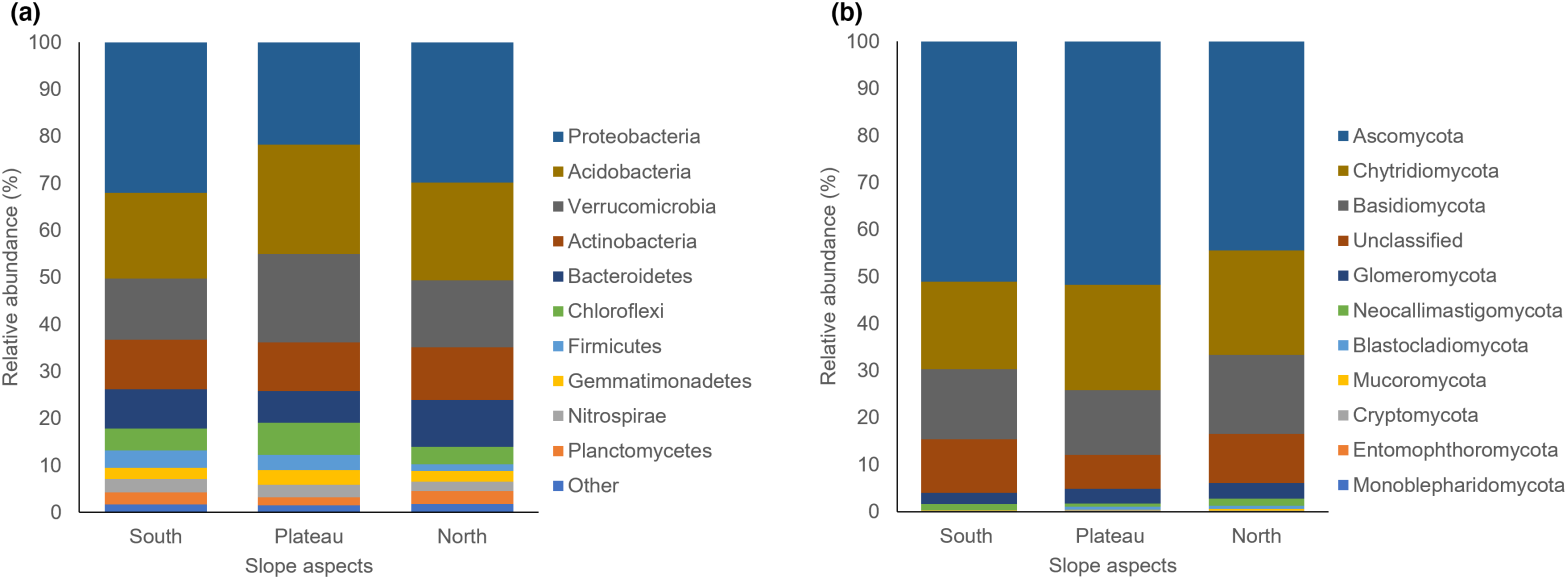
Relative abundance of bacterial and fungal phyla across the three montane aspects. Bar charts represent the most abundant (a) bacterial and (b) fungal sequences (>1%) classified at the phylum level per montane aspect. “Other” under bacterial phyla represents all other phyla with relative abundance <1%.

These numbers represent the average percentages of bacterial phyla across all nine sites on the plateau and the north‐ and south‐facing slopes. However, when comparing the different slope aspects across the summit, differences in phyla abundance are observed (Figure 3a). The microbial communities on the south‐ and north‐facing slopes incorporated proportionately more Proteobacteria (10.68% and 9.95%) than on the plateau (7.28%). By contrast, the plateau included a greater relative abundance of Acidobacteria (7.75%) and Verrucomicrobia (6.26%), compared to the north‐ (6.93% and 4.75%) and south‐facing slopes (6.06% and 4.35%), respectively.

Fungal community on the sampled mountain comprised of taxa belonging to 10 distinct phyla, along with one labeled as “general classification of Fungi,” 35 classes and 475 genera. The fungal soil microbiome across all three slope aspects was predominated by members of the phyla Ascomycota (49%, mainly from the classes Sordariomycetes – 12% of total, Leotiomycetes – 11%, Dothideomycetes – 7%, and Eurotiomycetes – 7%), Chytridiomycota (21%, all from the class Chytridiomycetes – 21%), and Basidiomycota (15%, mainly the class Agaricomycetes – 12% of total). The most abundant genera across the three slope aspects included *Mortierella* (9%), *Phlyctochytrium* (6%), *Leptodontidium* (4%), *Coniochaeta* (3%), and *Fusarium* (3%).

As with the bacteria, differences are noted between the sampled slope aspects (Figure 3b). The plateau fungal microbiome incorporated a higher relative abundance of Ascomycota (17.27%) and Chytridiomycota (7.46%), compared to the south‐ (17.02% and 6.22%) and north‐facing slopes (14.80% and 7.42%), respectively. The south‐facing slope lacked representatives of the phylum Monoblepharidomycota and a very low abundance of Blastocladiomycota (0.01%), compared to the north‐facing slope (0.01% and 0.22%) and the plateau (0.02% and 0.23%) where these were only found in certain replicates. Mucoromycota and Neocallimastigomycota abundances were marginally higher on the north‐facing slope (0.10% and 0.53%) compared to the south‐facing slope (0.04% and 0.44%) and plateau (0.02% and 0.24%), although all these phyla were found at relatively low abundance on each aspect.

### 3.3 Bacterial and fungal α‐diversity

**FIGURE 4:**
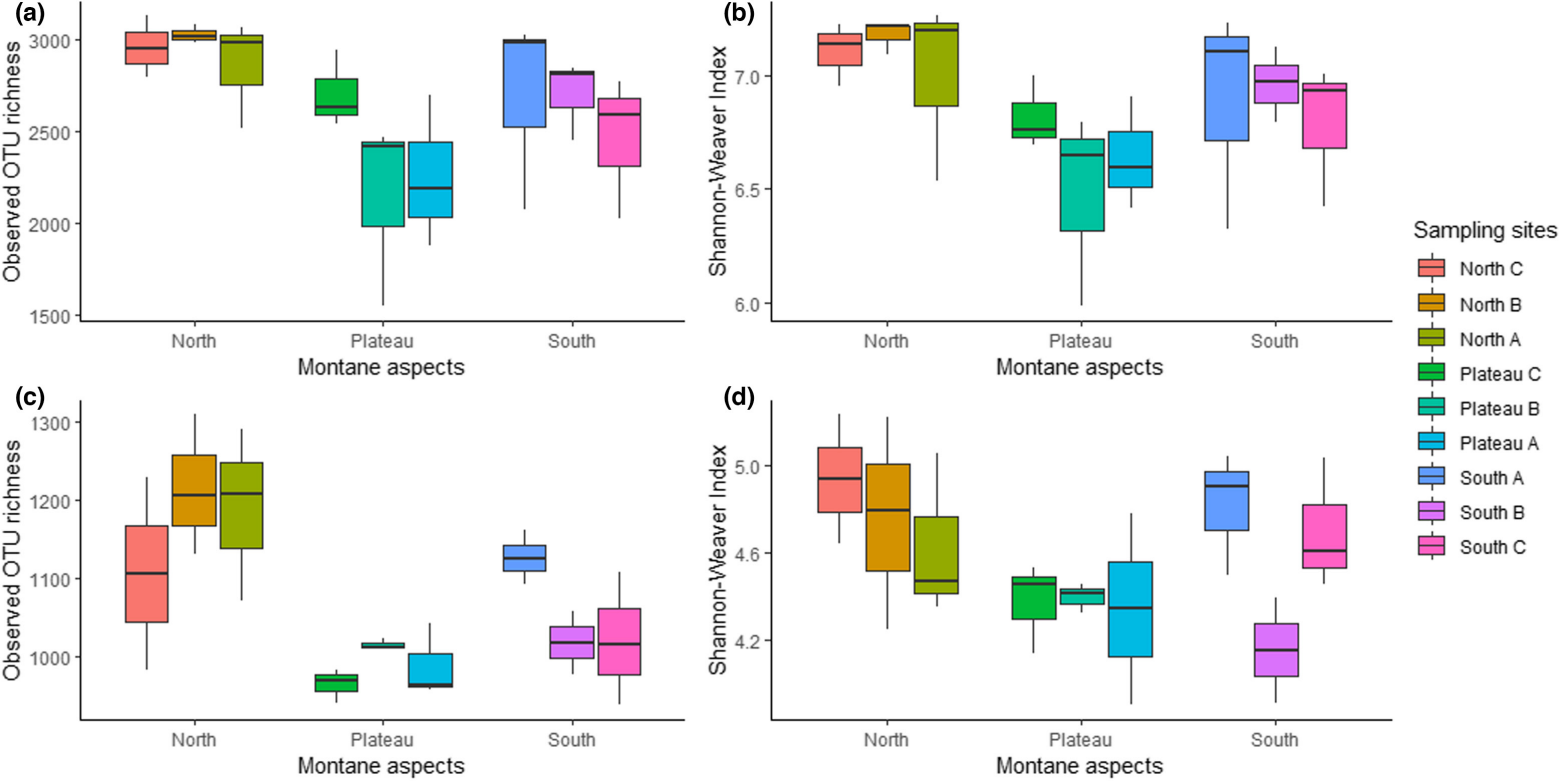
Variation in α‐diversity indices for bacterial and fungal communities on the sampled mountain. Boxplots represent the observed OTU richness and Shannon‐Weaver diversity indices for the different sampling points for the bacterial (a and b) and fungal counterparts (c and d), respectively. North A and South A were located at the top of the mountain followed by North B and South B in the middle and North C and South C at the bottom of the mountain slope. Plateau A represents the edge on the southern end Plateau C the edge on the northern end of the mountain, with Plateau B in the middle. Each sample point incorporates three replicates at each site, with the exception of NC and SB for fungi, where one replicate was eliminated due to low read counts.

To evaluate the diversity of bacterial and fungal species within each of the montane sampling sites, observed OTU richness and Shannon‐Weaver indices were determined (Table A2). Relatively greater bacterial diversity could be observed for the north‐facing slope compared to the south‐facing slope and plateau (Figure 4b). For both the north‐ and south‐facing slope, the highest diversity occurred at uppermost localities (NA and SA) closest to the rock scarps, followed by the middle (NB and SB) and lower localities (NC and SC), further away from the rock scarps. The highest diversity for the plateau was observed for the sample site (PC) nearest the uppermost sample site of the northern slope (NA). Similarly, bacterial observed OTU richness was higher on the north‐facing slope compared to the south‐facing slope, which decreased down the slope and on the plateau (Figure 6a).

To determine the impact of slope aspect on bacterial observed OTU richness and Shannon diversity, the non‐parametric equivalent of ANOVA, the Kruskal–Wallis test, was used. This was determined based on the Shapiro–Wilk test for normality, which indicated that bacterial data for both observed OTU richness (*W* = 0.91, *p*‐value = .018) and Shannon diversity (*W* = 0.91, *p*‐value = .027) followed non‐normal distribution (*p*‐vale < .050). A significant difference in bacterial observed OTU richness and Shannon diversity was noted between the plateau and the north‐ and south‐facing slopes using the Kruskal–Wallis test (Observed OTU richness: chi‐square = 10.31, *p*‐value = .006, Shannon diversity: chi‐square = 10.10, *p*‐value = .006, degrees of freedom (df) = 2). This indicated that the three slope aspects studied did have a significant impact on the diversity of bacteria across the sampled mountain. Differences between the various slope aspects and their impact on bacterial diversity were further determined using the Wilcoxon rank‐sum test. This analysis indicated that bacterial observed OTU richness was significantly different between the north‐facing slope and the plateau (*p*‐adjusted = .006) and between the north‐ and south‐facing slope (*p*‐adjusted = .047). A significant difference in the Shannon diversity was also noted between the north‐facing slope and the plateau (*p*‐adjusted = .008) only.

Apart from the middle of the south‐facing slope (SB), the lowest fungal diversity was observed across all three sampling points along the plateau (Figure 4d). Fungal diversity was also greater on the north‐facing slope compared to the south‐facing slope and plateau. In contrast to bacterial diversity on the north‐facing slope, the middle portion (NB) mid‐way from the rock scarp recorded the greatest fungal observed OTU richness, followed by the top (NA) and lower site (NC) sampling points which were closer toward and further away from the rock scarp, respectively. In contrast, fungal observed OTU richness decreased down the south‐facing slope (from SA to SC) with increasing distance from the rock scarp (Figure 4c). In contrast to the bacterial counterpart, the Shapiro–Wilk test used for fungal observed OTU richness (*W* = 0.93, *p*‐value = .080) and Shannon diversity (*W* = 0.96, *p*‐value = .325) indicated that these data were not significantly different from normality, and thus followed normal distribution (*p*‐value > .050). For the fungal dataset, ANOVA and Tukey HSD tests were used to determine the impact of slope aspect on observed OTU richness and Shannon diversity. A significant difference in fungal observed OTU richness was also noted for the various slope aspects (df = 2, *F*‐value = 13.51, *p*‐value = .0001) using ANOVA. By contrast, Shannon diversity was not significantly affected. The difference between the various aspects and their impact on observed OTU richness and Shannon diversity was further determined using Tukey HSD. This test indicated that fungal OTU richness was significantly different only between the north‐facing slope and the plateau (*p*‐adjusted < .005) and between the north‐ and south‐facing slope (*p*‐adjusted = .017), as with bacterial observed OTU richness.

A general linear model with Quasipoisson (log)‐ and Gaussian distribution was used to determine the impact of mean annual soil temperatures (MAST), mean annual relative humidity (MARH), slope aspect, and the distance of sampling locations from the rock scarps on the north‐ and south‐facing slope on bacterial and fungal diversity, respectively. The bacterial and fungal observed OTU richness and Shannon diversity were not significantly affected by changes in MAST and MARH in soil independently, and as interactions with slope aspect (*p*‐value > .050). This was despite slope aspect significantly affecting both MAST (df = 2, *F*‐value = 6.10, *p*‐value = .003) and MARH in soil (df = 2, *F*‐value = 9.21, *p*‐value < .005) across the plateau and the north‐ and south‐facing slopes, as determined by ANOVA and Tukey HSD. The distance of sampling locations from the rock scarps on the north‐ and south‐facing slopes also had no significant impact on bacterial and fungal observed OTU richness and Shannon diversity independently, and as interactions with MAST and MARH in soil (*p*‐value > .050). This was despite the visible zonation in vegetation and climatic conditions noted across the sampling locations on the north‐ and south‐facing slopes. Distance of sampling locations from the rock scarps itself did not play a significant impact on MAST (df = 2, *F*‐value = 0.118, *p*‐value = .889) and MARH in soil (df = 2, *F*‐value = 2.518, *p*‐value = .088).

### 3.4 Bacterial and fungal β‐diversity

The differences between bacterial and fungal soil microbial communities across the different slope aspects were determined using PERMANOVA based on Bray–Curtis dissimilarity. Bacterial community structure was significantly affected by slope aspect, MARH in soil, and distance of sampling locations from the rock scarps on the north‐ and south‐facing slopes independently (Table 2). Slope aspect, however, was found to have a greater influence as it accounted for higher variance (*R* ^2^) compared to the distance of sampling locations from the rock scarps on the north‐ and south‐facing slopes. Likewise, the effect of the interaction of MAST and MARH in soil with slope aspect and distance of sampling locations from rock scarps on the north‐ and south‐facing slopes on bacterial communities was less than that of slope aspect itself. These interactions were also found to have no significant influence on bacterial community structures on the summit.

**TABLE 2:** Effects of abiotic and geographical factors on bacterial and fungal community structures.

	Bacteria				Fungi			
	df	*F*‐model	*R* ^2^ (%)	*p*‐Value	df	*F*‐model	*R* ^2^ (%)	*p*‐Value
Slope aspect	2	3.77	23.92	**.001**	2	2.25	16.98	**.001**
MAST	1	1.46	5.52	.119	1	1.35	5.53	**.037**
MARH	1	2.51	9.12	**.004**	1	1.79	7.20	**.007**
Slope aspect × MAST	2	1.43	8.52	.081	2	1.33	9.58	**.022**
Slope aspect × MARH	2	1.35	8.23	.095	2	1.46	10.41	**.006**
Distance from rock scarp	1	1.94	10.82	**.021**	1	1.63	10.43	**.005**
Distance from rock scarp × MAST	1	1.59	7.92	.067	1	1.20	7.28	.112
Distance from rock scarp × MARH	1	1.21	6.18	.239	1	1.06	6.52	.315

*Note*: Table 2 shows the *F*‐ratios for the test conducted to assess the effects of abiotic (soil temperatures and relative humidity) and locational attributes (slope aspect and distance from rock scarp) on bacterial and fungal community structures. df indicates the degrees of freedom, and *R* ^2^ indicates the proportion of variation explained by the factors. Significant *p*‐values are indicated in bold. Permutations were taken at 1000.

Abbreviations: MAST, mean annual soil temperature; MSRH, mean annual relative humidity.

Fungal community structure, like its bacterial counterpart, was significantly affected by slope aspect, MARH in soil, and distance of sampling locations from rock scarps on the north‐ and south‐facing slopes independently (Table 2). Likewise, slope aspect had a greater influence with a higher variance compared to the distance of sampling locations from the rock scarp on the north‐ and south‐facing slopes independently, as determined using PERMANOVA. Furthermore, fungal community structures were significantly influenced by MAST on the summit independently. In contrast with bacteria, fungal community structures were significantly affected by the interactions of MAST and MARH in soil with slope aspect. The effect of these interactions was, however, less than the effect slope aspect on its own had on fungal community structures.

**FIGURE 5:**
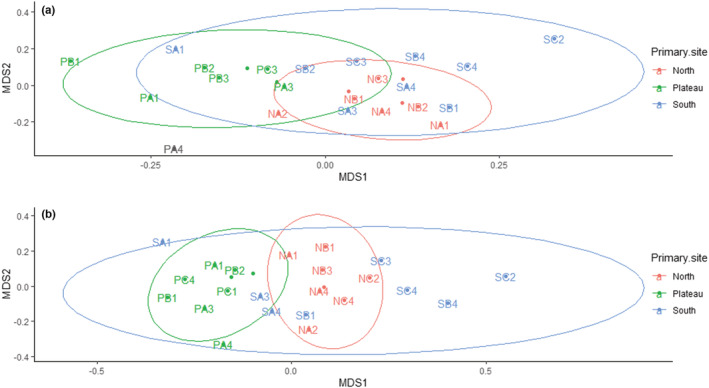
Bacterial and fungal community structures across all sample sites for the three montane aspects. nMDS plots represent (a) bacterial and (b) fungal community structures across the montane aspects based on Bray–Curtis distance. The distance between points is indicative of the relative dissimilarities in community structures, with the ellipses indicating dispersion regions of each site. Bacterial stress solution was reached at about 0.16 and fungal at 0.21, both of which were relatively low.

nMDS plots were used to visualize the community structures of both bacteria and fungi (Figure 5a,b). Bacterial communities across the different sample sites on the north‐facing slope clustered more closely together compared to the south‐facing slope and plateau and fully/partially overlapped with the south‐facing slope/plateau, respectively (Figure 5a). Variation in the bacterial communities was greatest along the south‐facing slope transect, with samples from the top (SA1), middle (SB4), and lower sites (SC2 and SC4) separated from the main cluster of the three slope aspects.

The effect of these interactions was, however, less than the effect slope aspect on its own had on fungal community structures. Similar to the bacterial community, fungal communities for all sample sites across the north‐facing slope were clustered. However, in the case of fungi, there was also an observable clustering of fungal communities across the plateau (Figure 5b). As with bacteria, fungal communities along the south‐facing slope also differed most strongly, with the same sample points along the lower (SC2 and SC4) and middle (SB4) sites separated from the main cluster of the three slope aspects.

Variations in bacterial and fungal community structure along the distinct montane aspects were further determined using beta dispersion. This analysis showed that there were no differences in the group variance of bacterial community structures (df = 2; *F*‐value = 0.90; *p*‐value = .416) between the plateau and the north‐ and south‐facing slopes. Pairwise comparisons also indicate no differences in variance between the north‐ and south‐facing slopes (*p*‐value = .122), the plateau and north‐facing slope (*p*‐value = .542), and the plateau and south‐facing slope (*p*‐value = .542). While there was no difference in the group variance of fungal community structures (df = 2, *F*‐value = 3.44, *p*‐value = .006), differences in the pairwise comparisons of variance were observed between the north‐ and south‐facing slopes (*p*‐value = .017), as well as the plateau and south‐facing slope (*p*‐value = .039). No differences were observed between the plateau and north‐facing slope (*p*‐value = .919). These would account for the overlaps and dispersion of replicates observed for the plateau and the north‐ and south‐facing slopes (Figure 5a,b).

### 3.5 Bacterial and fungal indicator taxa

As the slope aspect exhibited the strongest effect on bacterial and fungal communities, differential abundance testing using the DESeq2 approach was conducted to identify specific OTUs associated with a particular slope. The factors of interest used included the plateau compared to the north‐ and south‐facing slope and the south‐facing slope compared to the north‐facing slope given variations in topographic influence, and vegetation and climatic conditions. From this analysis, a total of 669 bacterial and 345 fungal OTUs were identified as significantly (*p*‐adjusted < .05) associated when comparing the plateau with the north‐ and south‐facing slope. In contrast, a total of 191 bacterial and 199 fungal OTUs were identified as significantly (*p*‐adjusted < .05) associated when comparing the south‐ and north‐facing slopes. These significantly associated OTUs were classifiable at the genus level.

**FIGURE 6:**
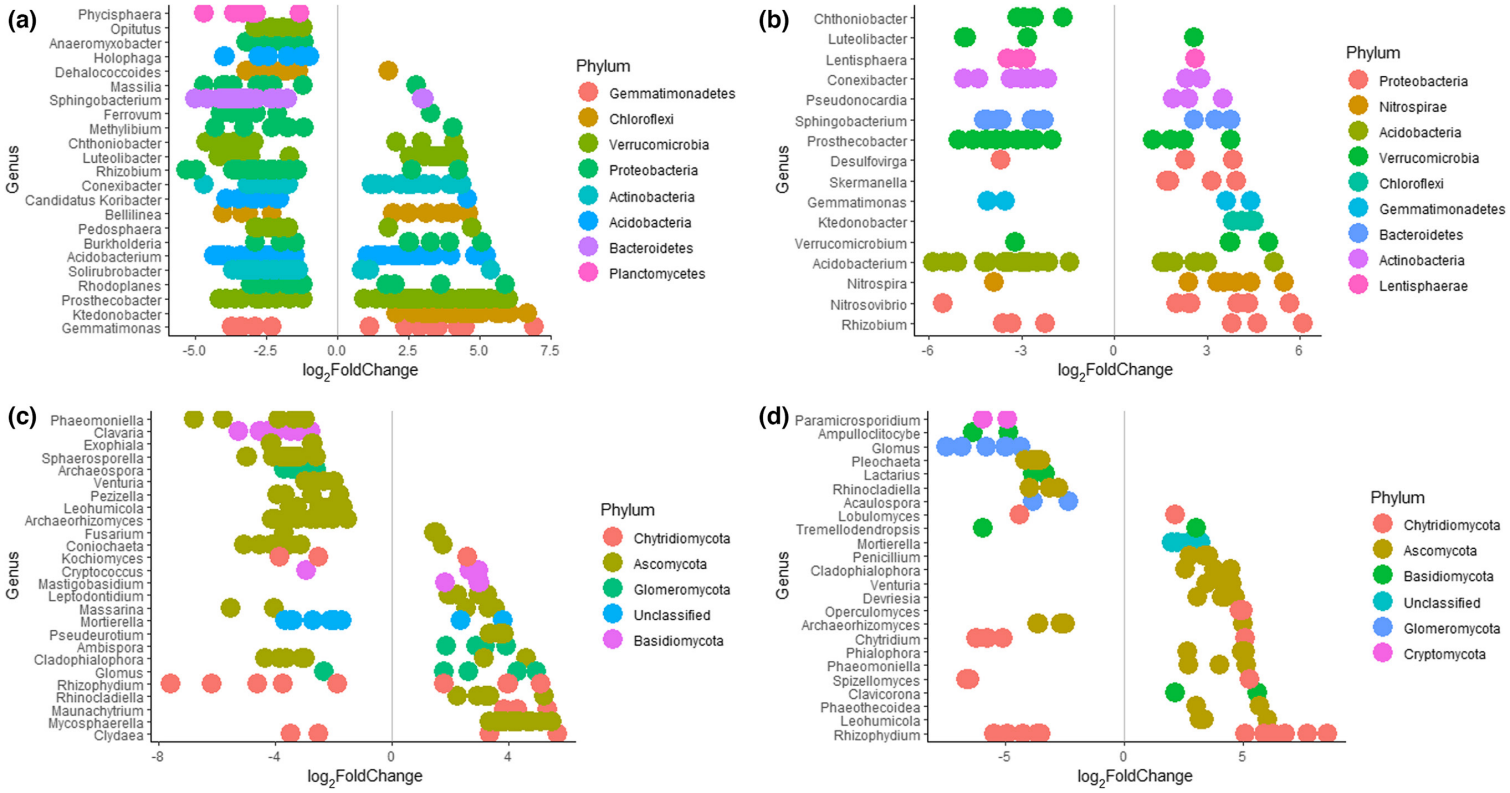
Bacterial and fungal phyla identified as indicators on the slope aspects. Dot plots represent the most abundant significant indicators (*p*‐adjusted < .050) of bacterial (a, b) and fungal (c, d) phyla classified at genus level. The target factors included the plateau (a, c) and south‐facing slope (b, d) in comparison to the base levels which included the north‐ and south‐facing slope and the north‐facing slopes, respectively. Prevalence of particular taxa for the target factors is represented by log_2_FoldChange > 0, in contrast to prevalence of particular taxa for the base levels is represented by log_2_FoldChange < 0. The dots represent the number of indicator OTUs present of each genus.

Bacterial taxa from the phyla Proteobacteria (191 OTUs, mainly from the genus *Rhizobium*), Actinobacteria (60 OTUs, mainly from the genus *Solirubrobacter*), Acidobacteria (47 OTUs), and Bacteroidetes (46 OTUs) were more prevalent across the vegetated north‐ and south‐facing slopes (log_2_FoldChange < 0) compared to the plateau (38 OTUs, 28 OTUs, 27 OTUs, and 9 OTUs), respectively (log_2_FoldChange > 0) (Figure 6a). Contrastingly, taxa from the phyla Verrucomicrobia (60 OTUs, mainly from the genus *Prosthecobacter*), Chloroflexi (38 OTUs, mainly from the genus *Ktedonobacter*), and Gemmatimonadetes (10 OTUs, mainly from the genus *Gemmatimonas*) were more prevalent across the warm bare soils of the plateau, compared to the north‐ and south‐facing slopes (41 OTUs, 18 OTUs, and 5 OTUs), respectively. Taxa from the phyla Verrucomicrobia (21 OTUs, mainly from the genus *Prosthecobacter*) were also more prevalent across the warmer north‐facing slope (log_2_FoldChange < 0) compared to the cooler south‐facing slope (11 OTUs) (log_2_FoldChange > 0) (Figure 6b).

Similarly, fungal taxa also show variations in distribution of indicator taxa across the three slope aspects. Fungal taxa from the phyla Basidiomycota (31 OTUs, mainly from the genus *Clavaria*) and Chytridiomycota (20 OTUs, mainly from the genus *Rhizophydium*) were more prevalent across the vegetated soils of the north‐ and south‐facing slopes (log_2_FoldChange < 0) compared to the plateau (16 OTUs and 14 OTUs), respectively (log_2_FoldChange > 0) (Figure 6c). Taxa from the phyla Basidiomycota (14 OTUs) and Glomeromycota (9 OTUs) were also more prevalent on the warmer north‐facing slope (log_2_FoldChange < 0) compared to the south‐facing slope (9 OTUs and 1 OTU), respectively (log_2_FoldChange > 0) (Figure 6d). Taxa from the phylum Ascomycota had the greatest prevalence compared to all the indicator taxa and were distributed widely across the north‐ and south‐facing slopes (142 OTUs, mainly from the genus *Sphaerosporella*) and the plateau (84 OTUs, mainly from the genus *Mycoshaerella*) (Figure 6c).

## 4 DISCUSSION

The Lesotho highlands have been the focus of a broad range of geo‐ and bio‐environmental studies covering disciplines, such as geology, geomorphology, botany, climate, hydrology, and environmental change (e.g., Carbutt & Edwards, 2004; Fitchett et al., 2016; Grab & Knight, 2018; Mapeshoane & van Huyssteen, 2016; Norström et al., 2018). However, to date, there has been no attention given to microclimate and its control on biodiversity in these mountains, and studies on soil microbial diversity and community structure have been completely absent in the region. To this end, we have here presented a metataxonomic study, with a view to establishing the impact of varying slope aspects on bacterial (16 S rRNA) and fungal (ITS) soil communities across a summit in the Lesotho highlands. A total of 1,750,536 sequences were obtained from the summit, with fungal taxa accounting for 65% of these.

Slope aspect of mountainous terrain is known to significantly control environmental and microclimatic conditions such as temperature and moisture, among a host of other soil properties (Wu et al., 2017). At our Drakensberg site, the three slope aspects of the selected summit significantly affected soil temperatures and RH, as noted between May 2019 and April 2020. Variations in soil temperatures and RH across the slopes are attributed to microsite differences in precipitation, snow cover accumulation and longevity, and exposure to insolation (Boelhouwers & Meiklejohn, 2002; Grab, 1999; Mills et al., 2009). Temperature and RH recorded for soils across the three slope aspects in our study were also likely affected by the presence of snow cover, in particular below the south‐facing rock scarp where snow tends to last longer (Grab et al., 2017).

Abnormally high RH recordings across the plateau and the north‐ and south‐facing slopes can be attributed to the formation of water films over the iButtons during periods of supersaturation associated with high precipitation events or rapid snow melt (Cáceres et al., 2007). The lasting occurrence of snow below the rock scarp at higher elevations on the south‐facing slope is likely the reason for the near‐constant temperature ranges and abnormally high RH readings between the winter months of May–August 2019. Snow accumulation further insulates soils from freezing temperatures, thereby protecting above‐ and below‐ground biota from extensive freezing during colder winter months (Contosta et al., 2019; Freppaz et al., 2007). The absence of shading and increased levels of solar radiation exposure on the plateau and the north‐facing slope would have contributed to rapid snow melt and greater soil exposure, hence a greater range in temperatures and RH recorded along such topographic portions of our transect. Such inferences are supported by previously published arguments (Edwards et al., 2007; Grab et al., 2017).

The most common bacterial phyla found on the plateau and the north‐ and south‐facing slopes included Acidobacteria, Actinobacteria, Bacteroidetes, Proteobacteria, and Verrucomicrobia, which accounted for more than 80% of all the bacterial phyla on the summit. These bacterial phyla also dominated soils across cryic biomes which are typical to high mountain regions of the world, including the Changbai and Shennongjia Mountains in China, the Italian Alps, and the Tibetan Plateau (Shen et al., 2013; Siles & Margesin, 2016; Yuan et al., 2014; Zhang et al., 2015).

While these bacterial phyla were predominant across the sampled summit, variations in their abundance could be observed along different slope aspects. A greater abundance of Acidobacteria, Chloroflexi, Gemmatimonadetes, and Verrucomicrobia was found across the plateau, together with a greater association of specific Chloroflexi, Gemmatimonadetes, and Verrucomicrobia indicator OTUs, compared to the north‐ and south‐facing slopes. The north‐ and south‐facing slopes in contrast had a greater abundance and association of specific Proteobacteria, particularly Alpha‐ and Betaproteobacteria and Bacteroidetes indicator OTUs. Apart from temperature and RH differences between slope aspects, variations may also be associated with the lifestyle of these microorganisms. Oligotrophic microorganisms such as Acidobacteria, Chloroflexi, Gemmatimonadetes, and Verrucomicrobia are able to survive under nutrient‐limited conditions (Ho et al., 2017; Tada et al., 1995; Yao et al., 2017), whereas copiotrophic microorganisms such as Proteobacteria require nutrient‐rich environments to survive (Koch, 2001; Yao et al., 2017). This compares favorably with our observations where the nutrient‐poor plateau is associated with generally desiccated thin soils and exposed bedrock with little to no plant cover, while the north‐ and south‐facing slopes had a diverse range of vegetation and soil coverage, accounting for more nutrient‐rich micro‐environments.

The fungal microbiome across the summit mostly comprised of the phyla Ascomycota, Basidiomycota, Chytridiomycota, Unclassified fungal taxa, and Glomeromycota, which accounted for 98% of all phyla. The presence of these fungal phyla has similarly been reported from the Taibai Mountain in China, montane forests in South America, and retreating glaciers in the North American Arctic Transect (Geml et al., 2014; Ren et al., 2018; Timling et al., 2014).

Fungi are known to play several essential roles in soil, such as nutrient cycling, decomposition of organic matter, and forming symbiotic relationships with plant species (Krishnamoorthy et al., 2015; Schoch et al., 2009; Wang et al., 2020). Alterations in the diversity and composition of fungal communities due to changes in environmental and climatic conditions could thus have an impact on ecosystem functioning and vegetation establishments in soil (Adamczyk et al., 2019). As with bacteria, variations in the abundance of these fungal phyla across the three slope aspects were noted. The soil microbiome of the plateau and north‐facing slope incorporated a greater abundance of Chytridiomycota, Basidiomycota, and Glomeromycota, compared to the south‐facing slope. However, indicator OTUs from these phyla appeared to be greatly associated with the north‐ and south‐facing slopes, compared to the plateau. Ascomycota prevailed across all three slope aspects in terms of relative abundance, as well as indicator OTUs. As with bacteria, variations can be attributed to lifestyle strategies, with certain saprotrophic fungi, such as Chytridiomycota, likely possessing characteristics of copiotrophic microorganisms due to their need for nutrient‐rich environments for decomposition (Ho et al., 2017; McConnaughey, 2014). In contrast, Glomeromycota and Basidiomycota may be classified as oligotrophic microorganisms (Ho et al., 2017). The presence of these phyla corresponds with their ability to survive in less vegetated and bare soil environments such as Drakensberg plateaus, as well as on the more vegetated north‐ and south‐facing slopes. The presence of copiotrophic microorganisms, including Chytridiomycota, on the plateau, may be indicative of other potential drivers of such phyla in soils across this region. These include but are not limited to vegetation types, soil temperatures, and soil properties such as nutrient composition, pH, and carbon and nitrogen compositions which are known to influence soil fungal communities (Zhou et al., 2021).

Members of the phylum Ascomycota may be classified as either copiotrophic or oligotrophic microorganisms, regardless of them being saprotrophic (Yao et al., 2017), such that the isolated genera *Fusarium* and *Penicillium* are oligotrophic microorganisms, while *Alternaria* and *Cladosporium* are copiotrophic microorganisms (Ho et al., 2017). This ability of various taxa within Ascomycota to utilize generalized lifestyle strategies stems from the capability of this phylum to better withstand environmental stressors and use a large variety of resources, thus allowing them dominate soils in greater abundance (Egidi et al., 2019). This corresponds with the presence of this phylum and their represented genera at high abundance with a greater association of indicator OTUs across all three mountain locations (aspects) investigated, regardless of the environmental conditions present. The presence of the rare fungal phylum, Neocallimastigomycota, albeit at relatively low abundance across the north‐ and south‐facing slopes (NB and SB), is most likely associated with livestock fecal matter. Although members of this phylum may be found as resistant structures in the soil at times, they are generally restricted to the digestive systems of herbivorous animals (Liggenstoffer et al., 2010).

Bacterial α‐diversity and community structure (β‐diversity) were significantly affected by slope aspect (north, south, and plateau) over what is a relatively short transect distance of approximately 170 m. Albeit over a greater sampling distance of just under 1000 m, Wu et al. (2017) similarly recorded bacterial diversity associated with slope aspect on Mt. Cardrona, New Zealand. The north‐facing slope of our Drakensberg summit had a greater bacterial and fungal diversity (observed OTU richness and Shannon diversity) than on the south‐facing slope and the plateau. The lower bacterial and fungal diversity on the plateau may be attributed to harsh environmental and microclimatic conditions (extreme wind, high levels of insolation, large temperature, and moisture fluxes).

Bacterial and fungal diversity on the north‐facing slope can be used as a possible indication (proxy) of (for) future projected climate warming across the south‐facing slope and other cooler regions of the Lesotho highlands. The north‐facing slope is substantially warmer and drier than the adjoining south‐facing slope. With continued warming and likely associated drying across the south‐facing slope, the possibility of shifts in microbial and plant species increases (Kerns et al., 2018). Species dependent on cooler habitats are more prone to shift to thermally suitable habitats over short distances across mountains (Scherrer & Körner, 2011). Furthermore, recent decadal‐scale changes in snowfall patterns, as is evident for the Lesotho highlands (Grab et al., 2017), may exert considerable influence on microbial species composition due to changes in the length of vegetation growing seasons (Scherrer & Körner, 2011). Ecological disturbances, which are expected in a warming climate, will inevitably affect microbial and plant species distribution, and thereby facilitate the transition to new community compositions of microbial and plant species (Kerns et al., 2018). With an ongoing warming scenario, it is assumed that: (1) microbial diversity and distribution on the south‐facing slope may gradually mirror those that are currently present on the north‐facing slope, and (2) the north‐facing slope microbial diversity and abundance may gradually decrease and more strongly mirror that of the plateau.

Apart from slope aspect, the summit in our study included the presence of a rock scarp along the south‐facing slope, thus contributing to notable microclimatic and vegetational zonation on the slope below. This topographic situation itself provides for the somewhat “unique” microclimate and environment below the rock scarps, hence further strengthening the slope‐aspect control on bacterial and fungal diversity and community composition.

## 5 CONCLUSIONS

Our study provides the first investigation of soil microbiome characteristics in the Drakensberg and Lesotho highlands of southern Africa. Differences in soil microbiomes over exceptionally complex but small spatial scales are attributed to topographic (slope aspect) and associated microclimatic controls. The soils across the summit in this study were found to contain highly diverse bacterial and fungal communities, despite the highly variable environmental conditions that were exhibited across the three slopes. Bacterial communities across the three slopes were predominated mainly by members of the phyla Acidobacteria, Proteobacteria, and Verrucomicrobia, while fungal communities were predominated by members of the phyla Ascomycota, Basidiomycota, and Chytridiomycota. Several bacterial and fungal phyla observed in this region are related to essential ecosystem processes and functions. The abundance of specific members of these phyla can thus be used as indicators of possible changes in environmental conditions based on the lifestyle strategies followed and essential environmental factors that act as drivers of changes in community composition. Slope aspect had a greater impact on bacterial and fungal diversity and community structure than did the distance of sampling location below a rock scarp. Despite this finding, the examined influence of the rock scarp provides new insight into previously unknown topographic controls affecting micro‐abiotic dynamics and associated soil microbiome characteristics. Overall, our research approach and findings from current warmer slopes provide the potential to project future changes on current cooler slopes, given assumed climate warming scenarios. We hope such a methodological design sets an example of how the “natural laboratory” might be used to project future microbiome changes within complex topographies subjected to climate warming.

## AUTHOR CONTRIBUTIONS

**Jasmin Patel:** Conceptualization (supporting); data curation (lead); formal analysis (lead); investigation (lead); methodology (supporting); software (lead); validation (equal); visualization (lead); writing – original draft (lead); writing – review and editing (lead). **Stefan Grab:** Conceptualization (supporting); data curation (supporting); formal analysis (supporting); funding acquisition (supporting); investigation (supporting); methodology (supporting); project administration (supporting); supervision (supporting); validation (supporting); visualization (supporting); writing – original draft (supporting); writing – review and editing (supporting). **Pieter De Maayer:** Conceptualization (lead); data curation (supporting); formal analysis (lead); funding acquisition (lead); investigation (supporting); methodology (lead); project administration (lead); resources (supporting); software (supporting); supervision (lead); validation (supporting); visualization (supporting); writing – original draft (supporting); writing – review and editing (supporting).

## FUNDING INFORMATION

This research was supported by the South African National Research Foundation (NRF). Author JP has received the NRF Masters Scholarship SFH180517331412.

## CONFLICT OF INTEREST STATEMENT

The authors have no relevant financial or non‐financial interests to disclose.

### OPEN RESEARCH BADGES

This article has earned Open Data and Open Materials badges. Data and materials are available at https://doi.org/10.5061/dryad.v6wwpzh0j.

## Data Availability

Raw data (fasta sequence sets and OTU tables) are available for download from Dryad via the https://doi.org/10.5061/dryad.v6wwpzh0j. Any additional data pertaining to the study can be requested from the corresponding author.

## APPENDIX 1

**TABLE A1:** Overview of the soil temperatures and relative humidity across the selected summit.

	Annual mean	Maximum	Minimum	Fluctuation	Mean spring	Mean summer	Mean autumn	Mean winter
*Temperatures (°C)*								
North A	10.83	39.55	−6.55	46.10	13.48	11.75	9.68	8.30
North B	8.43	28.61	−0.97	29.57	10.96	11.58	8.76	3.38
North C	9.35	30.62	−3.47	34.08	12.28	12.21	9.21	4.47
Plateau A	7.53	29.58	−3.47	33.05	10.77	10.69	6.94	2.38
Plateau B	8.52	30.61	−6.45	37.06	11.55	11.39	7.92	3.74
Plateau C	8.98	37.65	−7.96	45.61	12.02	11.52	8.17	4.62
South A	4.25	26.13	−10.06	36.18	5.65	11.25	5.56	−3.62
South B	6.53	28.56	−6.03	34.59	8.31	12.69	8.57	−1.23
South C	6.97	26.06	−5.04	31.09	9.28	12.67	8.51	−0.67
*Relative humidity (%)*								
North A	81.69	107.42	24.61	82.82	69.59	94.66	98.05	68.79
North B	86.49	111.07	6.32	104.75	90.09	87.25	54.42	96.33
North C	97.90	107.78	71.17	36.61	93.25	98.70	94.76	102.37
Plateau A	86.95	109.02	41.49	67.53	81.88	76.30	78.69	103.55
Plateau B	89.70	111.85	36.98	74.87	81.68	88.40	96.49	92.67
Plateau C	78.57	107.44	19.37	88.07	58.59	91.19	91.34	74.16
South A	97.01	106.82	61.10	45.72	95.15	94.85	95.07	100.79
South B	101.74	107.68	70.84	36.83	103.98	98.40	97.17	104.81
South C	100.46	110.61	10.06	100.55	103.87	88.91	104.28	105.65

**TABLE A2:** Bacterial and fungal α‐diversity indices computed for the sample replicates.

Replicates	Bacterial		Fungal	
	Observed	Shannon	Observed	Shannon
SA1	2061	6.31	1129	4.50
SA3	2981	7.22	1160	4.90
SA4	3024	7.10	1092	5.04
SB1	2852	7.15	1059	3.91
SB2	2827	6.98	Removed	Removed
SB4	2471	6.80	995	4.40
SC2	2019	6.42	1031	5.04
SC3	2571	6.92	944	4.60
SC4	2772	7.00	1079	4.45
Mean ± SE	2619.78 ± 124.25	6.88 ± 0.11	1061.13 ± 24.88	4.61 ± 0.14
PA1	2167	6.60	959	4.34
PA3	2693	6.92	965	3.90
PA4	1874	6.41	1038	4.78
PB1	1542	5.98	1015	4.46
PB2	2406	6.64	1021	4.42
PB3	2511	6.79	995	4.31
PC1	2974	7.01	973	4.12
PC3	2591	6.74	927	4.45
PC4	2495	6.69	963	4.53
Mean ± SE	2361.44 ± 145.63	6.64 ± 0.10	984 ± 11.88	4.37 ± 0.08
NA1	3014	7.26	1201	4.33
NA2	2516	6.54	1083	4.47
NA4	3089	7.21	1268	5.05
NB1	3108	7.22	1194	4.80
NB2	3077	7.20	1318	5.23
NB3	3002	7.09	1135	4.24
NC2	2938	7.14	976	4.64
NC3	2782	6.95	Removed	Removed
NC4	3134	7.22	1235	5.23
Mean ± SE	2962.22 ± 66.30	7.09 ± 0.08	1176.25 ± 38.59	4.75 ± 0.14

*Note*: Shown are the α‐diversity measures computed for bacteria and fungi. Sample nomenclature indicates the region from which the sample replicates were collected (N, north‐facing slope; P, plateau; S, south‐facing slope). Mean ± SE indicates the mean together with the standard error calculated per slope aspect.
